# Determination of Critical Nitrogen Dilution Curve Based on Stem Dry Matter in Rice

**DOI:** 10.1371/journal.pone.0104540

**Published:** 2014-08-15

**Authors:** Syed Tahir Ata-Ul-Karim, Xia Yao, Xiaojun Liu, Weixing Cao, Yan Zhu

**Affiliations:** National Engineering and Technology Center for Information Agriculture, Jiangsu Key Laboratory for Information Agriculture, Nanjing Agricultural University, Nanjing, Jiangsu, P. R. China; Zhejiang University, China

## Abstract

Plant analysis is a very promising diagnostic tool for assessment of crop nitrogen (N) requirements in perspectives of cost effective and environment friendly agriculture. Diagnosing N nutritional status of rice crop through plant analysis will give insights into optimizing N requirements of future crops. The present study was aimed to develop a new methodology for determining the critical nitrogen (N_c_) dilution curve based on stem dry matter (S_DM_) and to assess its suitability to estimate the level of N nutrition for rice (*Oryza sativa* L.) in east China. Three field experiments with varied N rates (0–360 kg N ha^−1^) using three Japonica rice hybrids, Lingxiangyou-18, Wuxiangjing-14 and Wuyunjing were conducted in Jiangsu province of east China. S_DM_ and stem N concentration (SNC) were determined during vegetative stage for growth analysis. A N_c_ dilution curve based on S_DM_ was described by the equation (N_c_ = 2.17W^−0.27^with W being S_DM_ in t ha^−1^), when S_DM_ ranged from 0.88 to 7.94 t ha^−1^. However, for S_DM_ < 0.88 t ha^−1^, the constant critical value N_c_ = 1.76% S_DM_ was applied. The curve was dually validated for N-limiting and non-N-limiting growth conditions. The N nutrition index (NNI) and accumulated N deficit (N_and_) of stem ranged from 0.57 to 1.06 and 51.1 to −7.07 kg N ha^−1^, respectively, during key growth stages under varied N rates in 2010 and 2011. The values of ΔN derived from either NNI or N_and_ could be used as references for N dressing management during rice growth. Our results demonstrated that the present curve well differentiated the conditions of limiting and non-limiting N nutrition in rice crop. The S_DM_ based N_c_ dilution curve can be adopted as an alternate and novel approach for evaluating plant N status to support N fertilization decision during the vegetative growth of Japonica rice in east China.

## Introduction

Estimating nitrogen (N) nutritional status is a key to investigating, monitoring, and managing cropping systems [1]. Conventional farming has led to extensive use of N as a tool for ensuring profitability in the soils with uncertain fertility levels, which has raised the concerns about environmental sustainability. A reliable diagnosis of crop N requirement and nutritional status give insight into optimization of qualitative and quantitative aspects of crop production. It also improve N use efficiency and add to environmental protection [2]. Soil and plant-based strategies are two principle approaches, extensively used to derive information about the N nutrition status of crops, for satisfying their demand for N and to minimize N losses [3]. The former rarely describes the intensity of N release over a longer period, so the latter are widely accepted and adopted. Therefore, the present study investigates a plant-based strategy for an in-season assessment of N nutrition status for rice crop.

In plant-based approaches, the N nutrition status is generally monitored to determine the requirement for top dressing in crops [3]. For this purpose, several plant-based diagnostic tools, such as critical N concentration (N_c_) approach, chlorophyll meter, hyper-spectral reflectance and remote sensing, have been successfully used for in-season N management [4]. They differ in scope, in context of reference spatial scale, in terms of monetary and time resources, as well as skills and expertise required for their implementation at field [5]. Despite being simple, chlorophyll meter readings are affected by leaf thickness, abiotic stress and nutrient variability [6]. Canopy reflectance method's accuracy is affected by solar illumination, soil background effects and sensor viewing geometry [4]. However, the concept of N_c_ can be used as a potential alternate to these techniques, and it can give insight into relative N status of a crop. The present study utilizes this concept for an in-season N fertilizer management in rice crop.

The concept of N_c_ is crop specific, precise, simple and biologically sound, because it is based on actual crop growth. Whole plant dry matter based N_c_ approach was successfully applied for N management in winter wheat [7], [8], corn [9] and spring wheat [10]. This approach was successfully applied for a Indica rice in tropics and Japonica rice in subtropical temperate region [11], [12]. Dry matter partitioning among different plant organs affects the weight/N concentration relationship, and changes the shape of the dilution curve, thus limits its acceptance as a reliable method [13], [14]. The concept of N_c_ for specific plant organs (e.g., leaves and stem) is similar to that on whole plant basis. Leaf based diagnosis of N status in crops is affected by progressive shading by newer leaves, decline of leaf N concentration due to aging, pest attack, abiotic stresses and increase in the proportion of structural tissues [15]. Stem sap nitrate concentration is influenced by phenological phase, cultivar, temperature and solar radiation [16]. During vegetative phase, the contribution of stem dry matter (S_DM_) towards total plant dry matter is significantly higher than that of leaf dry matter (L_DM_), hence it is the most determining factor for N dilution of the whole plant [17]. Thus, the idea of using N_c_ curve based on S_DM_ over whole plant dry matter and L_DM_ based methods, can be used as an alternate approach for determination of N_c_ dilution curve.

The objectives of this work were to develop a N_c_ dilution curve based on S_DM_ and to assess the plausibility of this curve to estimate N nutrition status of Japonica rice. The estimation based on this approach will be more reliable than existing methods due to consistency at different growth stages.

## Materials and Methods

### Ethics statement

The experiments land is owned and managed by Nanjing Agricultural University, Nanjing, China. Nanjing Agricultural University permits and approvals obtained for the work and study. The field studies did not involve wildlife or any endangered or protected species.

### Experimental details

Three field experiments with multiple N rates (0–360 kg N ha^−1^) were conducted using three contrasting Japonica rice hybrids, Lingxiangyou-18 (LXY-18), Wuxiangjing-14 (WXJ-14) and Wuyunjing (WYJ), at Yizheng (32°16′N, 119°10′E) and Jiangning (31°56′N, 118°59′E) located in lower Yangtze River Reaches of east China. The soil was clay loam and was classified as Ultisoles. The rice-wheat cropping system is practiced in the region. The applied N rates varied significantly among different farmers. The average rate of N fertilizer reached 387 kg ha^−1^ during the period of 2004–2008 [18].

The whole experimental area was ploughed and subsequently harrowed before transplanting. All bunds were compacted to prevent seepage into and from adjacent plots. A plastic lining was installed to a depth of 40 cm between drain and the bund of each plot to minimize seepage across the bunds towards the drains. To further minimize seepage of water from control plot (N_0_), double bunds were constructed separating them and the adjacent plots. Experiments were arranged in a randomized complete block design with three replications. The size of each experimental plot was 8 m by 4.5 m, with planting density of approximately 22.2 hills per m^2^. At site 1, soil pH, organic matter, total N, available phosphorous (P), and available potassium (K) were 6.2, 17.5 g kg^−1^, 1.6 g kg^−1^ 43 mg kg^−1^, 90 mg kg^−1^, and 6.4, 15.5 g kg^−1^, 1.3 g kg^−1^ 38 mg kg^−1^, and 85 mg kg^−1^ in 2010 and 2011, respectively. The corresponding soil properties were 6.5, 13.5 g kg^−1^, 1.13 g kg^−1^ 45 mg kg^−1^, 91 mg kg^−1^ in 2007 at site 2. For experiments conducted at site 1 in 2010 and 2011, treatment consisted of five N rates as 0, 80, 160, 240, and 320 kg N ha^−1^, and 0, 90, 180, 270, and 360 kg N ha^−1^, respectively, while for experiment conducted at site 2 in 2007, treatment consisted of three N rates as 110, 220, and 330 kg N ha^−1^. N in all experiments was distributed as 50% at pre planting, 10% at tillering, 20% at jointing, and 20% at booting, with urea as the N source. Aside from N fertilizer, phosphorus (135 kg ha^−1^) and potassium (190 kg ha^−1^) fertilizers were basally incorporated at the last harrowing and leveling in all plots before transplanting as monocalcium phosphate Ca(H_2_PO_4_)_2_ and potassium chloride (KCl). Rice seedlings at five leaves stage were transplanted in experimental fields on June 20 (site 1) in 2010 and 2011, and on 29 June (site 2) in 2007, respectively. Pre-emergence herbicides were used to control weeds at early growth stages. Also plots were regularly hand-weeded until canopy was closed to prevent weed damage. Insecticides were used to prevent insect damage. All other agronomic practices were used according to local recommendations to avoid yield loss.

### Sample collection and measurement

Rice plants were sampled from each plot at the intervals of 10–12 days from 0.23 m^2^ area (5 hills) at active tillering, mid tillering, stem elongation, panicle initiation, booting and heading stages during the period of each experiment for growth analysis. The plants were manually severed at ground level on each sampling date. Fresh plants were divided into green leaf blades and culm plus sheath. Samples were oven-dried at 105°C for half an hour to rapidly stop metabolism and then at 70°C until constant weight to obtain stem dry matter (S_DM_, t ha^−1^). The dried stem samples were ground and analyzed for total stem N concentration (SNC, %) by Kjeldahl method. Stem N accumulation (SNA, kg N ha^−1^) was obtained as summed product of the S_DM_ by the SNC. The SNC of whole-plant stem was calculated as SNA divided by S_DM_.

### Statistical analysis

The S_DM_ and SNC data for each sampling date, year and variety was separated and subjected to analysis of variance (ANOVA) using GLM procedures in SPSS-16 software package (SPSS Inc., Chicago. IL, USA). The differences among treatment means were measured by using the least significant difference (LSD) test at 90% level of significance, instead of classically used 95% in order to reduce the occurrence of Type II errors that could be high in such field experiments. For each measurement date, year and variety, the variation in the SNC versus S_DM_ across the different N levels was combined into a bilinear relation composed of a linear regression representing the joint increase in SNC and S_DM_ and a vertical line corresponding to an increase in SNC without significant variation in S_DM_. The theoretical N_c_ points corresponds to the ordinate of the breakout of the bilinear regression. Regression analysis was performed using Microsoft Excel (Microsoft Cooperation, Redmond, WA, USA).

### Construction and validation of critical, maximum and minimum N dilution curves

For determination of N_c_ dilution curve it is necessary to determine the N concentration that did not limit the S_DM_ production either by its excess or deficiency. The data used to construct the N_c_ dilution curve came from two experiments conducted in 2010 and 2011 by distinguishing the data points for N-limiting and non-N-limiting growth. The N-limiting growth treatment is defined as a treatment for which an additional N application leads to a significant increase in S_DM_. The non-N-limiting growth treatment is defined as a treatment, for which a supplement of N application does not lead to an increase in S_DM_ and, at the same time, exhibits a significant increase in SNC. If at the same measurement date, statistical analysis distinguished at least one set of N-limiting and non-N limiting data point, these data points were used either for construction of the N_c_ dilution curve or to validate it [7]. Consistent with earlier studies, an allometric function based on power regression (Freundlich model) was used to determine the relationship between the observed decreases in N_c_ with increasing S_DM._ The N_c_ dilution curve was validated first by using the data points not retained for establishing the parameters of the allometric function in 2010 and 2011, and then with independent data set from experiment conducted in 2007.

The data points (n = 13) from most plethoric N treatments (N_4_ plots) was assumed to represent the maximum N dilution curve (N_max_) while the minimum N dilution curve (N_min_) was determined by using the data points (n = 13) from the most N-limiting treatments for which N application was zero (N_0_ check plots).

### Calculation of critical N dilution curve based diagnostic tools

To identify the N status in the S_DM_ of rice during vegetative growth, the nitrogen nutrition index (NNI) and accumulated nitrogen deficit (N_and_) were established for each sampling date, experiment and variety. The NNI value was obtained by dividing the total N concentration of S_DM_ by N_c_ value determined by critical dilution curve, [9]. The N_and_ value for rice crop on each sampling date was obtained by subtracting the N accumulation under the N_c_ condition (N_cna_) from actual N accumulation (N_na_) under different N rates [12]. For in-season recommendation of supplemental N application, the difference value of NNI (ΔNNI), N_and_ (ΔN_and_) and difference value of N application rate (ΔN between different N treatments was calculated according to the method proposed by Ata-Ul-Karim et al. [12].

## Results

### Stem dry matter and nitrogen concentration

The S_DM_ production was significantly affected by N fertilization during the growth period of rice. The increase in S_DM_ followed a continuous increasing trend along with sampling dates for both the varieties during each year with increasing N rates from N_0_ to N_4_; however, there was no significant difference between N_3_ and N_4_ in all the cases (Table 1). This increase in the S_DM_ production with N fertilization may be linked to a higher absorption of N fertilizer. S_DM_ ranged from minimum 0.22 t ha^−1^ and 0.19 t ha^−1^ (N_0_) in WXJ-14 to a maximum of 7.22 t ha^−1^ and 8.04 t ha^−1^ (N_4_) in LXY-18 during 2010 and 2011, respectively. The results showed that there was no positive correlation between S_DM_ and N rates, as the S_DM_ tend to decrease when N rate exceeded a critical level. During each experimental year, S_DM_ conferred with the following inequality under different N ratess.
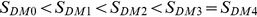
(1)where S_DM0_, S_DM1_, S_DM2_, S_DM3_ and S_DM4_ stands for S_DM_ of N_0_, N_1_, N_2_, N_3_ and N_4_, respectively.

**Table 1:** Changes of stem dry matter (S_DM_) with time (days after transplantation) under different N rates in two rice cultivars in experiments conducted during 2010 and 2011.

Year	Cultivar	DAT	Sampling date	Stem dry matter/Applied N (kg ha^−1^)					F prob.	LSD
				0	80	160	240	320		
**2010**	LXY-18	16	07-Jul	0.23	0.27	0.38	0.48	0.49	*	0.028
	LXY-18	26	17-Jul	0.63	0.78	0.95	1.11	1.12	*	0.055
	LXY-18	36	27-Jul	1.04	1.28	1.55	1.77	1.81	*	0.075
	LXY-18	48	08-Aug	2.23	2.73	3.25	3.61	3.51	*	0.226
	LXY-18	60	20-Aug	3.87	4.47	4.94	5.23	5.29	*	0.146
	LXY-18	70	30-Aug	4.72	5.56	6.7	7.01	7.22	*	0.279
	WXJ-14	16	07-Jul	0.22	0.27	0.32	0.36	0.35	*	0.019
	WXJ-14	26	17-Jul	0.39	0.54	0.73	0.9	0.91	*	0.045
	WXJ-14	36	27-Aug	0.55	0.8	1.13	1.38	1.46	*	0.063
	WXJ-14	48	08-Aug	1.22	1.65	1.99	2.18	2.23	*	0.11
	WXJ-14	60	20-Aug	2.77	3.46	3.72	4.19	3.97	*	0.233
	WXJ-14	70	30-Sep	3.69	4.4	5.04	5.81	5.7	*	0.205

				Stem dry matter/Applied N (kg ha^−1^)						
				0	90	180	270	360		
**2011**	LXY-18	18	09-Jul	0.22	0.33	0.4	0.56	0.59	*	0.042
	LXY-18	30	21-Jul	0.67	0.76	0.92	1.19	1.19	*	0.053
	LXY-18	42	02-Aug	1.12	1.28	1.42	1.78	1.76	*	0.132
	LXY-18	54	15-Aug	2.24	2.41	2.76	3.43	3.48	*	0.127
	LXY-18	64	25-Aug	3.59	4.02	4.37	4.75	4.85	*	0.164
	LXY-18	74	04-Sep	5.68	5.91	6.41	7.84	8.04	*	0.172
	WXJ-14	18	09-Jul	0.19	0.28	0.32	0.34	0.36	*	0.02
	WXJ-14	30	21-Jul	0.37	0.6	0.71	0.86	0.9	*	0.06
	WXJ-14	42	02-Aug	0.54	0.96	1.2	1.37	1.47	*	0.128
	WXJ-14	54	15-Aug	1.51	1.84	2.24	2.52	2.68	*	0.137
	WXJ-14	64	25-Aug	2.49	3.07	3.36	4.06	4.04	*	0.169
	WXJ-14	74	04-Sep	4.41	5.04	5.75	6.2	6.27	*	0.178

*: F statistic significant at 0.01 probability level.

**Figure 1:**
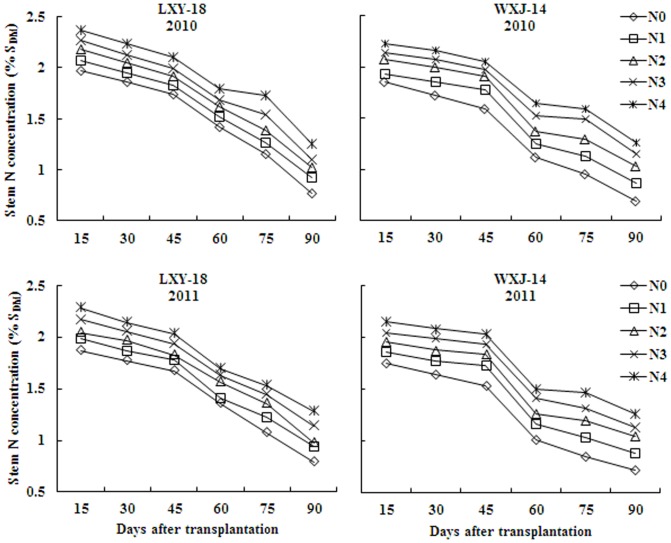
Changes of stem nitrogen concentration (% S_DM_) with time (days after transplantation) for rice under different N rates in experiments conducted during 2010 and 2011.

Stem N concentration response to N fertilizer rates was usually linear and a higher rate of N mostly resulted in a higher SNC, hitherto a decline in SNC was observed with increasing S_DM_ from active tillering to heading. Maximum variation in SNC of both cultivars was observed on 16 and 18 DAT, while minimum on 70 and 74 DAT, in years 2010 and 2011, respectively. The SNC ranged from 2.28 to 0.78 for LXY-18 and 2.16 to 0.71 for WXJ-14 during 2010, while 2.36 to 0.77 for LXY-18 and 2.23 to 0.68 for WXJ-14 during 2011 (Fig. 1).

### Determination of critical nitrogen dilution curves based on stem dry matter

**Figure 2:**
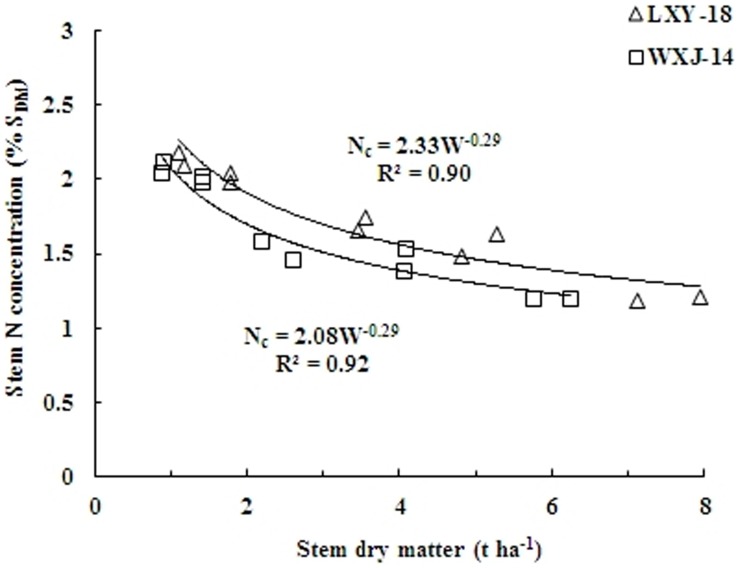
Critical nitrogen data points and N_c_ dilution curves in stem obtained by non-linear fitting for two rice cultivars (LXY-18, N_c_ = 2.33W^−0.29^ and WXJ-14, N_c_ = 2.08W^−0.29^) under different N rates in experiments conducted during 2010 and 2011.

A set of twenty theoretical data points for both cultivar, obtained from two experiments (10 data points for each cultivar) from active tillering to heading were used to calculate the N_c_ for a given level of S_DM_. The S_DM_ data that fit the statistical criteria for establishing N_c_ dilution curve varied from 0.88 t ha^−1^ to 7.94 t ha^−1^. A power functions were fitted to the calculated N_c_ points as equations (2) and (3), the coefficient for which were 0.90 and 0.92 for LXY-18 and WXJ-14, respectively (Fig. 2).
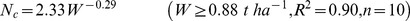
(2)

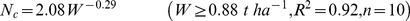
(3)where W is the S_DM_ expressed in t ha^−1^; N_c_ is the critical N concentration in stem expressed in % S_DM_; *a* and *b* are estimated parameters. The parameter *a* represents the N concentration in the S_DM_ when W = 1 t ha^−1^, and *b* represents the coefficient of dilution describing the relationship between N concentration and S_DM_.

The F-value (0.72) of two curves was less than the critical value of F_(1–18)_ = 4.41 at 5% probability level, showing non-significant difference between the curves [19], thus the data for the two varietal groups were united, and a unified dilution curve was determined as equation 4.
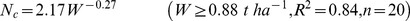
(4)


**Figure 3:**
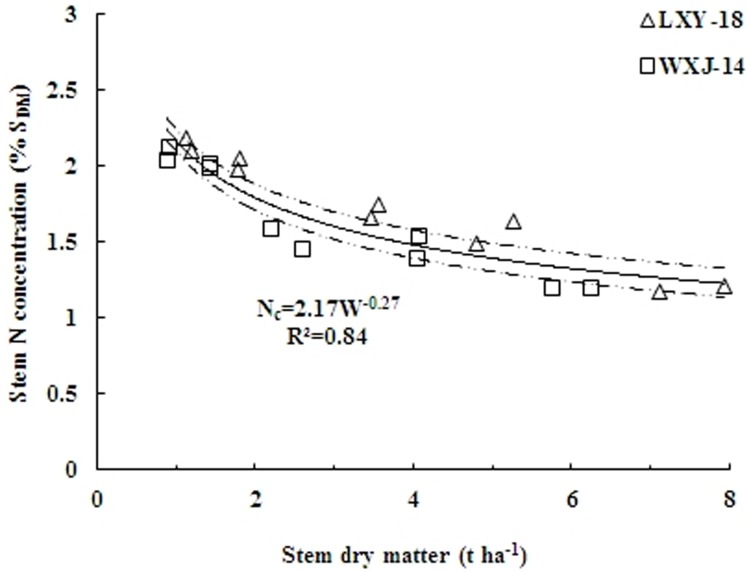
Critical nitrogen data points used to define the N_c_ dilution curve when data were pooled over for two rice cultivars (LXY-18 and WXJ-14). The solid line represents the N_c_ dilution curve (N_c_ = 2.17W^−0.27^; R^2^ = 0.84) describing the relationship between the N_c_ and stem dry matter of rice. The dotted lines represent the confidence band (P = 0.95).

The model accounted for 84% of the total variance. At early growth stages of rice crop, the N_c_ varied between 2.24% S_DM_ to 2.10% S_DM_ (95% confidence interval) for a S_DM_ of 0.88 t ha^−1^ at the lower end while 7.94 t ha^−1^ at the higher end, respectively (Fig. 3).

For the S_DM_ range of 0.1 to 0.88 t ha^−1^, corresponding to early growth stages, increasing N rates at sowing did not significantly affect S_DM_, because N requirement is relatively low during these early stages. Therefore, the N_c_ dilution curve cannot be applied to the low S_DM_<0.88 t ha^−1^ at early growth stages due to relatively smaller decline of N_c_ with increasing S_DM_. For these S_DM_, the N_c_ could not been determined by the same statistical method because the very high slope of the linear regression resulted in a highly variable estimate [7]. Hence, for the data points of S_DM_ ranging from 0.37 to 0.88 t ha^−1^ a constant N_c_ (1.76% S_DM_) was calculated as the mean value between the minimum N concentration of non-limiting N points (2.26% S_DM_) and the maximum N concentration of limiting N points (1.25% S_DM_), based on extrapolation of equation 4.

**Figure 4:**
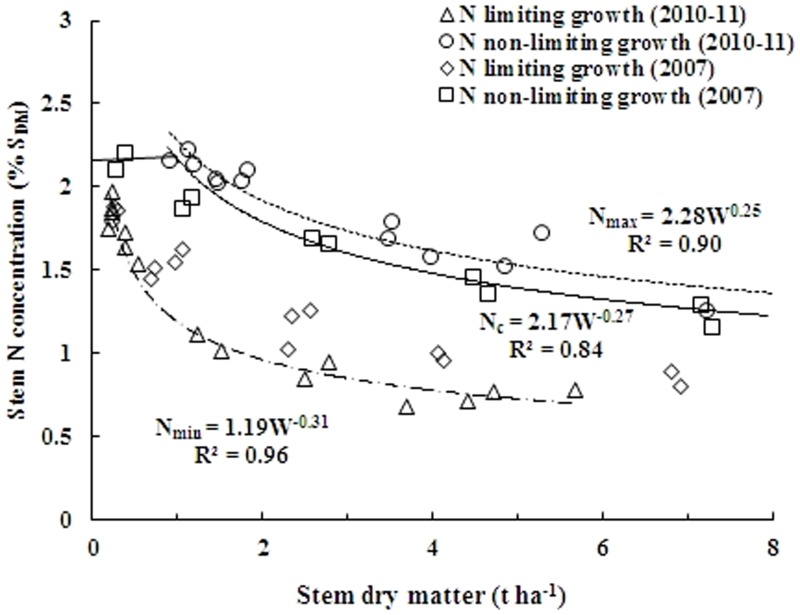
Comprehensive validation of N_c_ dilution curve using independent data set from experiment conducted in 2007. Data points (◊) represent N limiting growth conditions, while (□) represent N non-limiting conditions. The solid line in the middle represents the N_c_ curve (N_c_ = 2.17W^−0.27^) describing the relationship between the N_c_ and stem dry matter of rice. The data points (Δ) and (○) not engaged for establishing the parameters of allometric function (2010 and 2011) were used to develop two boundary curves, (–•–•–•) minimum limit curve (N_min_ = 1.19 W^−0.31^) and (------) maximum limit curve (N_max_ = 2.27W^−0.25^).

The above S_DM_ based N_c_ dilution curve was dually validated for N-limiting and non-N-limiting situations within the range for which it was developed. First, the curve was partially validated by combining the data points not engaged for establishing the parameters of the allometric function. In addition, the comprehensive validation of the curve was performed by using the data points from an independent experiment conducted in 2007. The results revealed that the N concentration data that led to the highest significant yields in S_DM_ were positioned close to or above the N_c_ dilution curve and considered to be non-N-limiting concentrations, whereas the data for the lowest significant S_DM_ yields, were positioned close to or under the N_c_ dilution curve and classified as N limiting values (Fig. 4). To determine N_max_, data points were selected only from non-N-limiting treatments (n = 13), and for N_min_, data points were selected from the treatment without N application (n = 13). Thus, the present N_c_ dilution curve could well discriminate the N limiting and non-N-limiting growing conditions in this study

### Changes of NNI and N_and_

**Figure 5:**
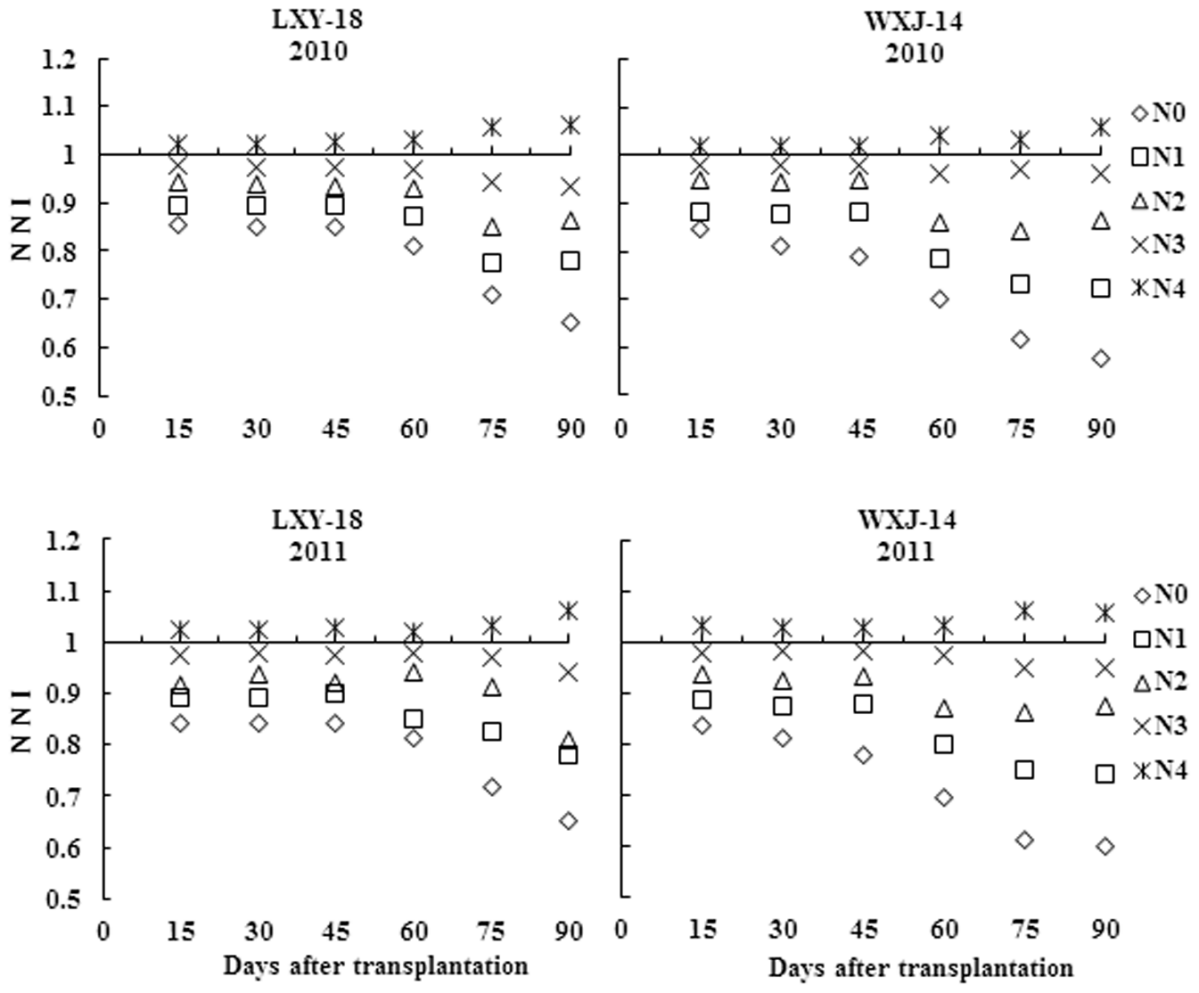
Changes of nitrogen nutrition index (NNI) with time (days after transplantation) for rice stem under different N rates in experiments conducted during 2010 and 2011.

**Figure 6:**
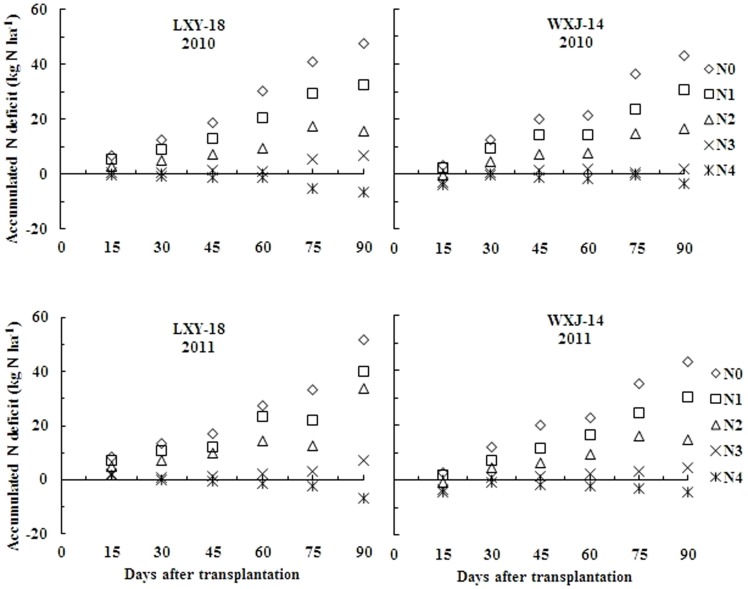
Changes of accumulated N deficit (N_and_) with time (days after transplantation) for rice stem under different N rates in experiments conducted during 2010 and 2011.

Nitrogen nutrition index and N_and_ are helpful in determining the crop nutrition status i.e. deficient, optimal or excess of N nutrition. N nutrition is considered as optimum when NNI = 1 and N_and_ = 0, while NNI>1 and N_and_<0 indicates luxury consumption of N nutrition, values of NNI<1 and N_and_>0 represents N shortage. NNI and N_and_ can be used to quantify the intensity of the N stress after the onset of N deficiency. Our results of significant differences in NNI and N_and_ across the growing seasons, N rates, and phenological stages in rice are in agreement with earlier reports for maize and wheat [10]. As seen in Figure 5 and 6, during 2010 and 2011 the NNI ranged from 0.65 to 1.06 for LXY-18 and 0.57 to 1.06 for WXJ-14, while the N_and_ ranged from 51.1 kg ha^−1^ to −7.07 kg ha^−1^ for LXY-18 and 43.3 kg ha^−1^ to −4.5 kg ha^−1^ for WXJ-14. The results showed that NNI amplified while N_and_ declined with increasing N rates, while both intensified steadily with growth of rice crop and reached to peaks at heading stage for N_0_, N_1_, N_2_ and N_3_ (N limiting treatments), nevertheless, for N_3_ this intensification was minor. In contrast, surplus N nutrition existed till heading stage for N_4_ (non-N-limiting treatment). The estimates based on NNI and N_and_ can be used to identify the N nutritional status at any stage of rice growth, allowing us to assess whether the N fertilizer dosage was ample enough to obtain higher yield in practice. These results confirmed the plausibility of using NNI and N_and_ to assess the status of N nutrition in rice plants growing under various conditions and stages.

**Figure 7:**
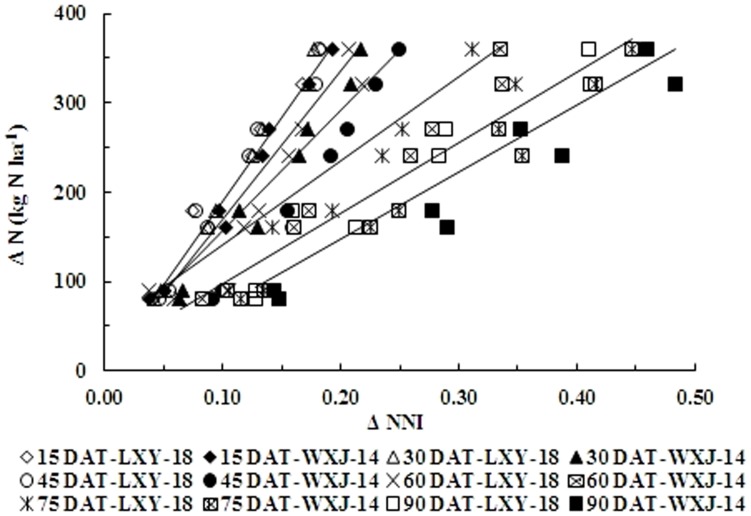
Relationship between changes of nitrogen nutrition index (ΔNNI) and changes of nitrogen application rates (ΔN, kg N ha^−1^) at different growth stages in experiments conducted during 2010 and 2011. The open symbols represent different growth stages for LXY-18 while filled symbols represent different growth stages for WXJ-14. (ΔN = A×ΔNNI+B; A = −16.60×DAT+2101, R^2^ = 0.95; B = −0.024×DAT^2^+2.57×DAT−40.07, R^2^ = 0.62).

**Figure 8:**
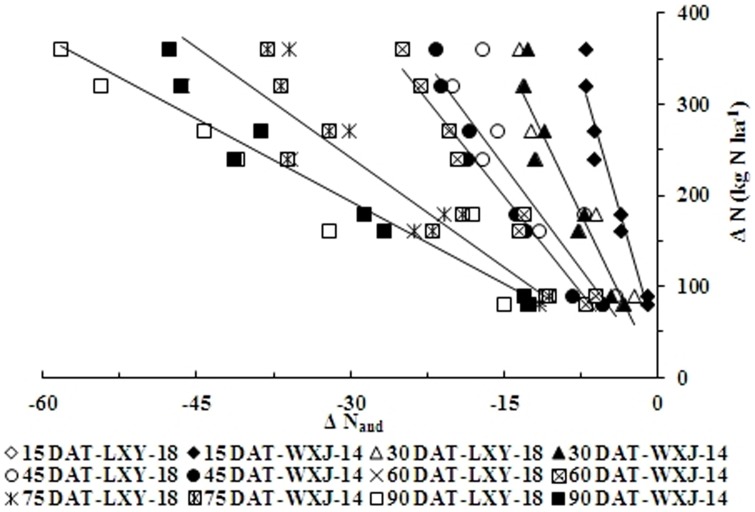
Relationship between changes of accumulated N deficit (ΔN_and_) and changes of nitrogen application rates (ΔN, kg N ha^−1^) at different growth stages in experiments conducted during 2010 and 2011. The open symbols represent different growth stages for LXY-18 while filled symbols represent different growth stages for WXJ-14 (ΔN = C×ΔN_and_+D; C = 18.97 ln(DAT)-89.22, R^2^ = 0.98 and D = −9.98 ln(DAT)+52.76, R^2^ = 0.19), respectively.

Figure 7 and 8 showed that ΔN had a positive correlation with ΔNNI and ΔN_and_. The simple linear regression equation showed non-significant differences between two varieties, although noticeable differences were observed among different phenological stages. Therefore, ΔN during growth period for both varieties could be derived from ΔNNI and ΔN_and_, respectively, according to the equations 5 & 6 as follows:
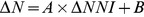
(5)

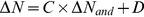
(6)


The parameters A, B, C, and D could be calculated from days after transplanting (DAT) using the equations as:
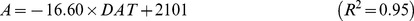
(7)

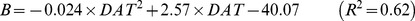
(8)

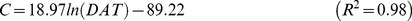
(9)

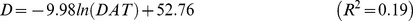
(10)


The ΔN obtained on the basis of relationship between ΔNNI, ΔN_and_ and ΔN, allowed us to make corrective decisions of N dressing recommendation for the precise N management during or even before the period of highest demand of the rice crop.

## Discussion

Application of N fertilizer for crop production is an economically viable option in terms of low cost as compared to the value of the marketable agricultural products themselves; however, N usage cannot assure a significant increase in crop productivity due to diminishing returns after certain levels. There is an increasing demand by strategy makers for simple-to-use, technically established and economically viable N indicators, which may allow monitoring and assessment of policy measures and offer tools for farm N management. With the advent of technology, more emphasis should be put on plant-based indicators, which simultaneously reflect the interactions between the plant and the soil. So far, there have been several reports on estimating the N_c_ concentration on whole plant dry matter basis in various crops, including rice [11], [12], and on L_DM_ basis in rice [20], yet no attempt was made to determine the N_c_ dilution curve on S_DM_ basis for any crop including rice. The current study has developed a S_DM_ based N_c_ dilution curve for rice in east China, thus providing a new approach for diagnosing and regulating N in crop species.

### Minimum and maximum nitrogen dilution curves

An obvious variability in SNC for a given range of S_DM_ was observed when all the data from three year experiments were analyzed for interpretation. This variability in SNC towards maturity of rice crop in present study was in agreement with earlier studies on winter wheat [7] and Japonica rice [12], and this variability could be attributed to a decline in the fraction of total plant N associated with photosynthesis [21], change in leaf/shoot ratio and self-shading of leaves [8].

Two boundary curves for N maximum (N_max_) and minimum (N_min_) have been determined by using maximum and minimum N concentration in S_DM_ and can be represented as equations:
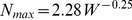
(11)

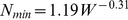
(12)


The N_max_ curve corresponding to the maximum N uptake in the S_DM_ without interfering with productivity and it can be considered as the first assessment of a maximum N dilution on S_DM_ basis in crops, and can be obtained with increasing N rates for maximum growth and N accumulation. This curve is an estimate of the maximum N accumulation capacity of stem which is regulated by mechanism associated with the growth and availability of soil N directly or indirectly via N metabolism [22]. The N_max_ curve in the present study shows a luxury consumption of N under N_4_ treatment, when N concentration exceeds N_c_ dilution curve and S_DM_ does not increase with increasing N rate. In contrast, the N_min_ curve is considered as a lower limit at which the N metabolism would soon stop to function. It corresponds to the minimum N taken up by rice plants under N_0_ treatment in present study. Thus, the N_min_ were used as the threshold concentration for proper metabolic functionality of the plant.

Moreover, the value of parameter *b* for the N_max_ was not significantly different from that of N_c_ dilution curve, which indicate that the partitioning of dry matter remains relatively constant when N uptake exceeds the N_c_ dilution curve. This is consistent with the concept of N_c_ dilution curve, which represents the lowest N at which maximum dry matter accumulation occurs. This implies that under luxury consumption of N, when N exceeds N_c_ dilution curve, dry matter accumulations does not increase with N and hence, dry matter partitioning will have similar value of parameter *b*. In contrast, for N_min_ curve under N stress, the value for parameter *b* tended to be slightly lower than the dilution curve. The relatively low value for *b* was associated with a change in dry matter partitioning.

### Comparison with other critical nitrogen dilution curves

**Figure 9:**
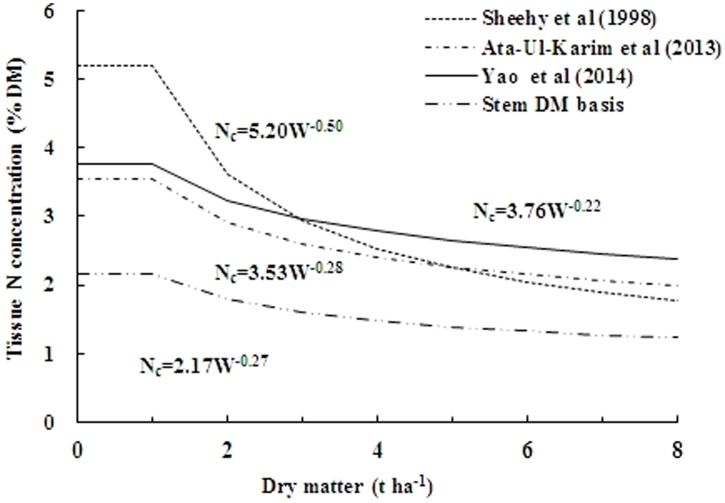
Comparison of different N_c_ dilution curves. The (------) represents the N_c_ dilution curve of Sheehy et al. (1998) (N_c_ = 5.20W^−0.50^) on plant dry matter basis in Indica rice under tropic environment. The (–•–•–•) represents the N_c_ dilution curve of Ata-Ul-Karim et al. (2013) (N_c_ = 3.53W^−0.28^) on plant dry matter basis in Japonica rice in Yangtze River Reaches. The (——) line represents N_c_ dilution curve of Yao et al. (2014) (N_c_ = 3.76W^−0.22^) on leaf dry matter basis in Japonica rice in Yangtze River Reaches, and the (–••–••–) line represents N_c_ dilution curve on stem dry matter basis in present study (N_c_ = 2.17W^−0.27^).

The concept of N_c_ dilution curve on whole plant dry matter and L_DM_ basis have already been successfully implicated for several crops including rice, yet no attempt was made to construct a S_DM_ based N_c_ dilution curve in any crop including rice. Figure 9 showed that the parameter *a* of N_c_ dilution curve on S_DM_ basis with Japonica rice developed in present study (2.17) was lower than the reference curve on whole plant dry matter basis of Indica rice in tropics (5.20) by Sheehy et al. [11] as well as lower than the curves developed with Japonica rice on whole plant dry matter basis (3.53) by Ata-Ul-Karim et al. [12], and L_DM_ basis (3.76) by Yao et al. [20].

The differences observed between the parameter *a* of dilution curve developed in present study and the curves on whole plant dry matter basis [11], [12] were due to morphological aggregation of structural components, which relates to the weight/N concentration in the whole plant [13]. Stress responses may cause differences in the partitioning of dry matter among various plant organs, and thereby affect the shape of the dilution curves. Moreover, dissimilarities in climatic conditions and genetic differences of Indica and Japonica rice contributed to the differences between the curves. The ability of Indica to hold higher plant N content and total N uptake [23]–[25] and faster growth rate [26], compared with those of Japonica rice, also lead to the differences between N_c_ dilution curve of Sheehy et al. [11] and that described in the present study. The differences of S_DM_ based curve with that of L_DM_ based one [20] are mainly attributed to leaf/stem ratio, because decrease in stem N during vegetative phase is related to decline in the metabolic biomass with high N contents, and increase in proportion of structural and non-photosynthetic biomass with low N contents [8]. Thus, higher proportion of structural biomass in stem than in leaves is responsible for the differences between the L_DM_ and S_DM_ based curves of Japonica rice.

The parameter *b* of the dilution curve indicates the dilution intensity of N during growth and the higher values of *b* indicate lower N dilutions [17].The coefficients *b* were (−0.50, −0.28, −0.22 and −0.274) for N_c_ dilution curve of Indica rice and for Japonica rice based on whole plant dry matter, L_DM_, and on S_DM_, respectively. The observed differences between the coefficients *b* of Indica rice and current S_DM_ based dilution curve might be explained by the differences in duration of vegetative phase in tropical and subtropical climates, while the differences between coefficients *b* of the curves of Japonica rice based on L_DM_ and S_DM_ were directly related to the distribution of dry matter between green leaves and the stem [17]. In contrast, the differences between coefficients *b* of the curves of Japonica rice on whole plant dry matter basis compared with that of S_DM_ basis, are negligible due to the reason that stem have a dilution effect on the N in the above ground tissues, because of their higher weight percentage in the total dry matter [27]. Therefore, the S_DM_ based dilution curve can be used as a potential alternative for in-season estimation of plant N nutrition status, instead of existing whole plant dry matter and L_DM_ based approaches.

### Implication for nitrogen diagnosis

The application of the present N_c_ dilution curve as a diagnostic tool for accurate N management to make corrective decisions of N dressing recommendation during rice production is very interesting. The N_c_ dilution curve can be used for a priori analysis intended to optimize fertilizer N management or for a posteriori diagnosis intended to detect N limiting nutrition for rice within experimental trials or fields in production. The a priori diagnosis of plant N status consists of timely detection of plant N deficiency during the crop growth cycle to determine the necessity of applying additional N fertilizer. Present study showed that the N_c_ dilution curve, resulting NNI and N_and_ effectively distinguished conditions of deficient, optimal and surplus N nutrition in rice. The values of ΔN in present study obtained on the basis of relationship between ΔNNI, ΔN_and_ and ΔN, permitted us to make corrective decisions of N dressing recommendation for precise N management during or even before the period of peak demand of the rice crop. The main limitation in using the present NNI and N_and_ directly as diagnostic tools is the need to determine the actual dry matter and N concentration, which can be monitored by the non-destructive means including remote sensing [28]–[30]. Moreover, a good correlation between these analytical tools and chlorophyll meter readings was previously reported by [9]. These indirect methods could possibly be a substitute for assessing NNI and N_and_ and portray crops and environments in conditions where they cannot be measured directly [31]. Thus, the models of NNI and N_and_, based on N_c_ dilution curve in relation to actual growth status, can be exploited directly for the estimation of crop N status to recommend the necessities of further N application during plant growth. These novel algorithms can also be combined into crop growth and management models to forecast crop N status and quantify N dressing plan. Although, NNI and N_and_ calculated in present study distinguished well the N-limiting and non-N-limiting growth conditions, a more comprehensive validation using different N management practices, N availabilities and cultivars is mandatory to robustly confirm the reliability of NNI and N_and_ usage as an investigative indicators for different ecological regions and rice production systems.

## Conclusions

In conclusion, we found that N fertilization endorses increase in the S_DM_, which was influenced by variations in SNC. A higher rate of N fertilizer generally increased SNC in Japonica rice; however, towards advancing maturity this increase followed a declining trend under different N levels, sampling dates and growing seasons. S_DM_ during vegetative growth period ranged from minimum value of 0.19 (N_0_) in WXJ-14 to a maximum value of 8.04 (N_4_) in LXY-18, whereas SNC varied from 0.68% in WXJ-14 to 2.36% in LXY-18 on S_DM_ basis under different N rates and growth stages. A new N_c_ dilution curve on S_DM_ basis for Japonica rice grown in east China was developed and can be described by equation, N_c_ = 2.17W^−0.274^, when S_DM_ ranges from 0.88 and 7.94 t ha^−1^, however for S_DM_<0.88 t ha^−1^, the constant critical value N_c_ = 1.76% S_DM_ was applied, which was independent of S_DM_. Additionally, the values of NNI and N_and_ at different sampling dates for N limiting condition were generally <1 and >0, while >1 and <0, respectively for non-N-limiting supply. The values of ΔN derived on the basis of relationship between ΔNNI, ΔN_and_ and ΔN, can be used to make corrective decisions of N dressing recommendation for precise N management, prior to or on the onset of the period of highest demand of the rice crop. We conclude that the S_DM_ based dilution curve developed in the present study offers a new vision into plant N status and can possibly be adopted as an alternate practical tool for reliable diagnosis of plant N status to correct N fertilization decision during the vegetative growth of rice in east China.
